# New insights into the regulation of innate immunity by caspase-8

**DOI:** 10.1186/s13075-015-0910-0

**Published:** 2016-01-13

**Authors:** Vitaliya Sagulenko, Kate E. Lawlor, James E. Vince

**Affiliations:** School of Chemistry and Molecular Biosciences, The University of Queensland, Brisbane, QLD 4072 Australia; Walter and Eliza Hall Institute, Parkville, Melbourne, VIC 3052 Australia; Department of Medical Biology, The University of Melbourne, Parkville, Melbourne, VIC 3050 Australia

## Abstract

Caspase-8 is required for extrinsic apoptosis, but is also central for preventing a pro-inflammatory receptor interacting protein kinase (RIPK) 3–mixed lineage kinase domain-like (MLKL)-dependent cell death pathway termed necroptosis. Despite these critical cellular functions, the impact of capase-8 deletion in the myeloid cell lineage, which forms the basis for innate immune responses, has remained unclear. In a recent article in *Arthritis Research & Therapy*, Cuda et al. report that myeloid cell-restricted caspase-8 loss leads to a very mild RIPK3-dependent inflammatory phenotype. The presented results suggest that inflammation does not arise exclusively because of RIPK3-mediated necroptotic death but that, in the absence of caspase-8, RIPK1 and RIPK3 enhance microbiome-driven Toll-like receptor-induced pro-inflammatory cytokine production.

The recent article by Cuda et al. published in *Arthritis Research & Therapy* [1] characterizes abnormal immune responses in mice lacking caspase-8 in myeloid cells. This research advances our understanding of caspase-8 function in monocytes, macrophages and neutrophils, which has been difficult to study because of the embryonic lethality of whole organism caspase-8 deficiency.

Caspase-8 is a cysteine–aspartic acid protease noted for its critical role in inducing death receptor and Toll-like receptor (TLR)-mediated apoptosis [2]. Caspase-8 is expressed as a monomeric zymogen, which requires dimerization and cleavage to achieve full activity. Upon ligand binding, death receptors such as Fas and tumour necrosis factor receptor 1 (TNFR1) recruit the adaptor protein Fas-associated death domain (FADD) that serves as a docking platform for pro-caspase-8 molecules and enables caspase-8 dimerization and transactivation. Active caspase-8 subsequently initiates apoptosis via proteolytic activation of downstream effector caspases. Caspase-8 activation has therefore been suggested as a promising approach for developing new anti-cancer therapies [3]. On the other hand, caspase-8 mutation causes lymphoproliferative disorder and immunodeficiency in people, and reduced caspase-8 expression is associated with atopic dermatitis and epidermal wound responses [4].

Recent mouse studies have uncovered additional roles for caspase-8 in regulating cell death and inflammatory responses, which may help explain its capacity to either induce or limit inflammation under specific conditions (Fig. 1). In some circumstances, TLR or death receptors can activate pro-inflammatory interleukin (IL)-1β, either via direct caspase-8-mediated proteolytic cleavage [5] or by caspase-8 activation of the Nod-like receptor 3 (NLRP3)–caspase-1 inflammasome [6]. Moreover, caspase-8 also acts in a pro-survival capacity, because its catalytic activity is essential for suppressing TLR and death receptor-mediated necroptotic killing [7]. When caspase-8 function is compromised, TLRs or TNFR1 signal through the receptor interacting protein kinase RIPK1 to induce the oligomerization and kinase activity of RIPK3. Subsequently, RIPK3 phosphorylates mixed lineage kinase domain-like (MLKL), and MLKL then inserts into cellular membranes, including the plasma membrane, to disrupt their integrity. Unlike apoptotic cell death, necroptosis-induced cellular rupture is thought to trigger inflammation through the release of intracellular danger molecules, termed damage-associated molecular patterns (DAMPs). Consistent with this, genetic inactivation of caspase-8 can signal necroptosis to cause inflammation, and necroptotic killing has been linked to a variety of diseases, including atherosclerosis and renal ischaemic reperfusion injury [7].

**Fig. 1:**
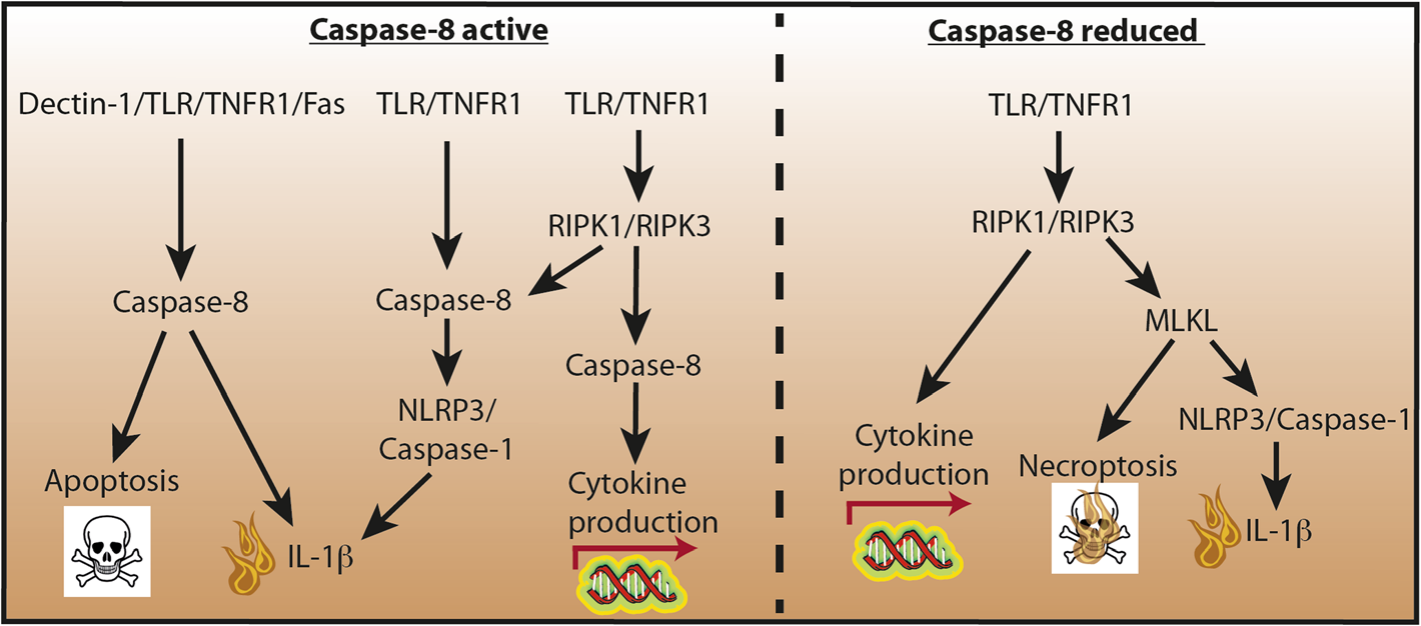
Inflammatory responses caused by caspase-8 activation, or deletion, in innate-immune cells. (*Left*) Other than inducing apoptosis, when activated through TLR, dectin-1 or death receptor signalling, caspase-8 has been reported to activate IL-1β through either the NLRP3–caspase-1 inflammasome or by direct proteolytic IL-1β processing. RIPK3 and caspase-8 signalling downstream of TLR4 is also required for efficient cytokine production independent of their ability to induce cell death. (*Right*) When caspase-8 is lost, TLR or TNFR1 ligation can activate RIPK1/RIPK3 to induce cytokine production, drive RIPK3–MLKL-dependent necroptosis (and DAMP release) or activate the NLRP3 inflammasome to generate bioactive IL-1β. *IL* interleukin, *MLKL* mixed lineage kinase domain-like, *NLRP3* Nod-like receptor 3, *RIPK* receptor interacting protein kinase, *TLR* Toll-like receptor, *TNFR1* tumour necrosis factor receptor 1

Macrophage survival during differentiation was previously reported to require caspase-8 expression [8], consistent with its role in keeping RIPK3-induced necroptosis signalling in check. However, Cuda et al. [1] now report relatively efficient caspase-8 deletion in neutrophils, monocytes and macrophages (Casp8^fl/fl LysMCre^). These mice developed mild systemic inflammation, presenting with splenomegaly and lymphadenopathy, increased splenic monocytes/macrophages, and a slight serological elevation in a subset of cytokines and chemokines. Strikingly, antibiotic treatment rescued the splenomegaly and lymphadenopathy in Casp8^fl/fl LysMCre^ mice, implying that intestinal microflora trigger the abnormal immune responses following myeloid cell-specific caspase-8 loss. Similarly, co-deletion of RIPK3 in Casp8^fl/fl LysMCre^ mice restored all inflammatory markers to normal. This implies that caspase-8 deletion alone is insufficient to trigger spontaneous RIPK3 signalling, but that upon microbe-dependent TLR engagement, caspase-8 blunts aberrant RIPK3 activity.

The tissue or cell-type specific deletion of caspase-8 has frequently been observed to induce a strong pro-inflammatory phenotype that is rescued by RIPK3 co-deletion [7]. Although DAMP release from dying cells is presumed to be the major inflammatory trigger, activated RIPK1/3 may also promote transcriptional cytokine responses [9,10]. Consistent with the latter, Cuda et al. observed that resting, M1 polarized or TLR-stimulated caspase-8-deficient macrophages displayed a distinct transcriptional profile, which was restored upon RIPK1 inhibition. These data imply that cell-intrinsic RIPK1/3-mediated gene induction contributes to the phenotype of Casp8^fl/fl LysMCre^ mice, independent of necroptotic DAMP release.

Caspase-8 deletion in dendritic cells (DCs) sensitizes mice to lipopolysaccharide (LPS)-induced lethality resulting from excess RIPK3-driven NLRP3 inflammasome activation and IL-1β production [11]. Similarly, caspase-8 deletion in the skin or gut triggers severe inflammation that is dependent on RIPK3 [12]. Somewhat contrary to expectations, Cuda et al. demonstrate that the injection of LPS into Casp8^fl/fl LysMCre^ mice results in reduced cytokine production compared with wild-type controls. This infers that caspase-8 may be required for optimal TLR-induced cytokine transcriptional responses, as observed previously in LPS-treated caspase-8 and RIPK3 doubly-deficient mice and macrophages [13]. The reduced cytokine production also suggests that LPS-triggered IL-1β activation could depend on caspase-8 activation of NLRP3–caspase-1, or on direct caspase-8 proteolysis of precursor IL-1β. Alternatively, it is also possible that LPS-induced necroptosis of caspase-8-deficient myeloid cells in vivo may blunt their capacity for producing inflammatory cytokines.

In summary, the research by Cuda et al. expands our knowledge of the multifaceted roles of caspase-8 in regulating immune responses. Although results point towards increased RIPK3 signalling causing aberrant TLR responses following caspase-8 loss in myeloid cells, it remains unclear how much of the in vivo phenotype is driven by necroptosis versus RIPK3-induced inflammatory cytokine production. In this regard, the co-deletion of the requisite downstream necroptotic protein MLKL in caspase-8-deficient myeloid cells may be particularly informative, because this would leave much of the RIPK1/3 inflammatory signalling capacity intact. Notably, the phenotype observed in myeloid cell-deficient caspase-8 mice contrasts with results observed upon caspase-8 deletion in DCs, which induces a more pronounced autoimmune phenotype that is not fully RIPK3 dependent [11,14]. This serves to highlight immune cell-type specific roles for caspase-8 that are critical for shaping both innate and adaptive immune responses.

## Abbreviations

DAMP: Damage-associated molecular pattern
DC: Dendritic cell
FADD: Fas-associated death domain
IL: Interleukin
LPS: Lipopolysaccharide
MLKL: Mixed lineage kinase domain-like
NLRP3: Nod-like receptor 3
RIPK: Receptor interacting protein kinase
TLR: Toll-like receptor
TNFR1: Tumour necrosis factor receptor 1
